# Evaluation of functional and structural leg length discrepancy in patients with adolescent idiopathic scoliosis using the EOS imaging system: a prospective comparative study

**DOI:** 10.1186/s13013-018-0152-4

**Published:** 2018-04-20

**Authors:** Tatsuhiro Sekiya, Yoichi Aota, Katsutaka Yamada, Kanichiro Kaneko, Manabu Ide, Tomoyuki Saito

**Affiliations:** 1 Department of Orthopedic Surgery, Yokohama City University, Fukuura 3-9, Kanazawa-ku, Yokohama City, Kanagawa Prefecture 236-0004 Japan; 2 Department of Orthopedic Surgery, Yokohama City Brain and Spine Center, Takigasira 1-2-1, Isogo-ku, Yokohama City, Kanagawa Prefecture 235-0012 Japan

**Keywords:** Adolescent idiopathic scoliosis, Leg length discrepancy, Spinopelvic parameters, EOS radiography

## Abstract

**Background:**

To our knowledge, no studies have reported the exact structural leg length discrepancies (LLDs) in patients with adolescent idiopathic scoliosis (AIS). Therefore, this study aimed to evaluate the differences between functional and structural LLDs and to examine the correlations between LLDs and spinopelvic parameters in patients with AIS using an EOS imaging system, which permits the three-dimensional reconstruction of spinal and lower-limb bony structures.

**Methods:**

Eighty-two consecutive patients with AIS underwent whole-body EOS radiography in a standing position between August 2014 and March 2016. Functional LLD, lumbar Cobb angle, thoracic curve Cobb angle, coronal balance, and pelvic obliquity were measured using two-dimensional EOS radiography. Structural LLDs were measured using three-dimensional EOS-reconstructed images. The comparison between LLDs was assessed using paired *t* test. Pearson’s correlation coefficient (*r*) was used to determine potential correlations between the LLDs and spinopelvic alignment parameters.

**Results:**

Functional LLDs were significantly larger than structural LLDs (5.6 ± 5.0 vs. 0.2 ± 3.6 mm, respectively; *p* < 0.001). Both functional and structural LLDs were significantly correlated with pelvic obliquity (*r* = 0.69 and *r* = 0.51, respectively; *p* < 0.001 for both). Functional LLD, but not structural LLD, was correlated with lumbar Cobb angle (*r* = 0.44, *p* < 0.001; *r* = 0.17, *p* = 0.12, respectively). In addition, functional and structural LLDs were not correlated with thoracic Cobb angle (*r* = 0.09 and *r* = − 0.05, respectively; *p* ≥ 0.68 for both).

**Conclusions:**

Although patients with AIS often have functional LLDs, structural LLDs tend to be smaller. The correlation between functional LLDs and the lumbar Cobb angle indicates that functional LLDs compensate for the lumbar curve. Thus, the difference between functional and structural LLDs indicates a compensatory mechanism involving extension and flexion of the lower limbs.

## Background

Adolescent idiopathic scoliosis (AIS) is a three-dimensional (3D) spinal deformity consisting of lateral deviation and axial rotation of the spine [1]. Clinical interest in 3D imaging for understanding, quantifying, and predicting the evolution of spinal deformities has long existed [2]. However, the gold standard for the initial diagnosis and longitudinal surveillance of AIS involves two-dimensional (2D) posterior-anterior (PA) full-length spine radiography [3].

**Fig. 1:**
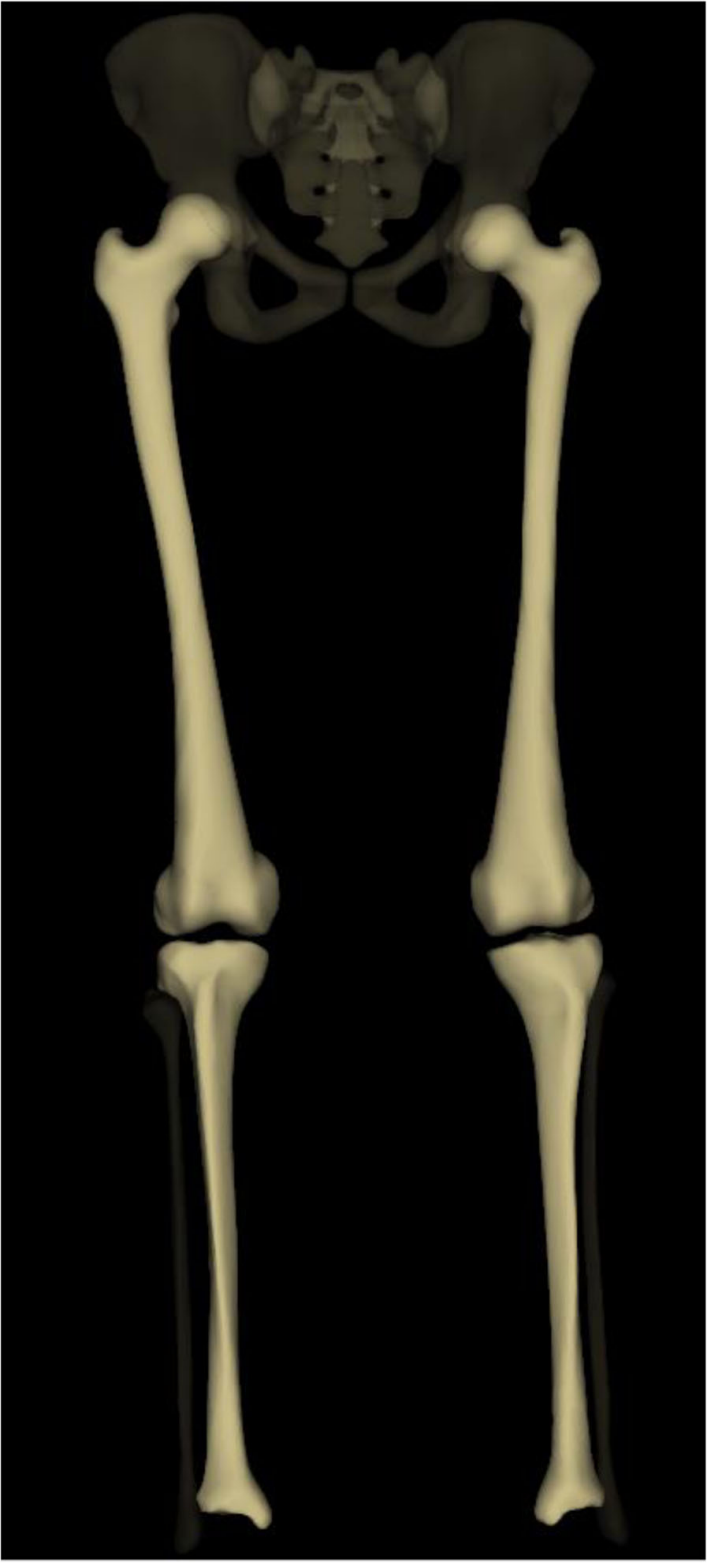
Three-dimensional imaging of the lower limbs using the EOS system. The EOS system was developed to accurately measure leg length three-dimensionally

In clinical practice, standing PA radiographs often reveal leg length discrepancies (LLDs) in patients with AIS. LLDs can be subdivided into functional LLDs, resulting from altered mechanics, and structural LLDs, which is associated with bony shortening [4]. Measuring the difference in femoral head heights on standing PA radiographs is a simple and reliable method for functional LLD documentation [5]. Unfortunately, limitations in measuring leg length using 3D imaging have been reported [6]. To our knowledge, no study has reported the exact structural LLDs in patients with AIS. In the past decade, a biplanar low-dose X-ray device, the EOS 2D/3D system (Biospace Imaging, Paris, France), was developed [7] to accurately measure leg length three-dimensionally (Fig. 1) [8]. In this study, we aimed to evaluate the differences between functional and structural LLDs and determine whether there are true LLDs in patients with AIS. In addition, we aimed to examine the correlations between LLDs and spinopelvic parameters in patients with AIS using the EOS imaging system.

## Methods

### Patients

Eighty-two consecutive patients with AIS (age range, 10–18 years; 70 girls and 12 boys) and Cobb angles of > 10° who were admitted to our hospital between August 2014 and March 2016 were surveyed. Patients with a history of scoliosis surgery, congenital scoliosis, secondary scoliosis, neuromuscular primeval scoliosis, scoliosis associated with intellectual disability, and/or congenital heart disease were excluded.

### Measurements

**Fig. 2:**
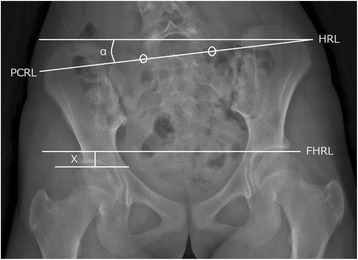
Functional leg length discrepancy and pelvic obliquity assessed using two-dimensional standing radiograph. Functional leg length discrepancy (X) and pelvic obliquity (α) measured using the two-dimensional posterior-anterior standing radiograph obtained from the EOS imaging system. FHRL femoral horizontal reference line, HRL horizontal reference line, PCRL pelvic coronal reference line

**Fig. 3:**
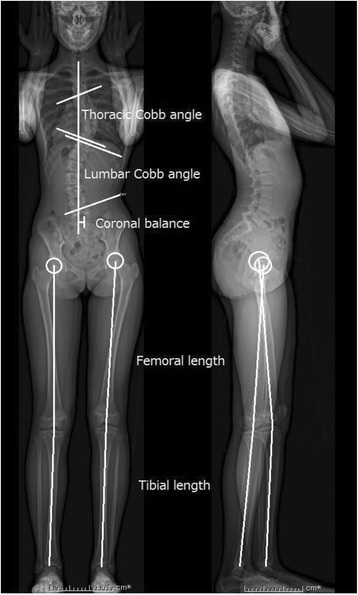
Measurements obtained using whole-body EOS radiography in the upright position. Thoracic Cobb angle, lumbar Cobb angle, and coronal balance were assessed in the coronal plane obtained from whole-body EOS radiography. Structural leg length discrepancy was defined as the difference between the sums of the bilateral femoral and tibial lengths obtained from three-dimensional distance using the EOS system

Patients underwent whole-body EOS radiography in the upright position during their first visit. We measured the functional LLD, lumbar Cobb angle, thoracic curve Cobb angle, pelvic obliquity, and coronal balance using 2D EOS PA standing radiographs, conducted according to the Spinal Deformity Study Group Radiographic Measurement Manual [9]. The femoral horizontal reference line was defined as a horizontal line tangent to the top of the highest portion of the femoral head. The height between right and left femoral horizontal reference lines was defined as the functional LLD. The angle between the horizontal reference line and the pelvic coronal reference line was defined as the pelvic obliquity. The tips of the sacral ala were used to create the pelvic coronal reference line (Fig. 2). Coronal balance was defined as the distance between the C7 plumb line and the center sacral vertical line, and a negative sign denotes a shift to the left. Structural LLD was defined as the difference between the sums of bilateral femoral lengths and tibial lengths obtained from three-dimensional distance using the EOS system. We took points with a front view and a side view and calculated a three-dimensional distance. The femoral length was measured from the femoral head to the intercondylar fossa, and the tibial length was measured from the intercondylar fossa to the inferior articular surface of the tibia (Fig. 3).

Additionally, the Risser sign, an indirect measure of skeletal maturity, was used as a bone growth parameter. Risser sign stage (0–5) was determined for each participant.

### Statistical analysis

Functional and structural LLDs were compared using a paired *t* test. Pearson’s correlation coefficient was used to investigate the correlations between the LLDs (functional and structural) and spinopelvic alignment parameters (pelvic obliquity, lumbar Cobb angle, and thoracic curve Cobb angle). A *p* value < 0.05 was considered to be statistically significant. Analyses were performed using Statistical Package for the Social Sciences, version 16.0 (SPSS, Chicago, IL, USA).

## Results

Patient demographics are presented in Table 1. Ten patients were classified according to Risser sign staging as stage 0, 5 as stage 1, 14 as stage 2, 15 as stage 3, 33 as stage 4, and 5 as stage 5. Of 58 female patients who experienced menarche, the mean age at menarche was 12.1 ± 1.2 years. According to the Lenke classification of AIS [10], type 1 AIS was noted in 50 patients, type 2 in 7 patients, type 3 in 9 patients, type 4 in 0 patients, type 5 in 16 patients, and type 6 in 0 patients.

**Table 1:** Demographic data

Characteristic	Value
Sex	
Male	12
Female	70
Age (years)	13.7 ± 1.9
Height (cm)	157.0 ± 7.4
Weight (kg)	45.4 ± 6.7
Seated height (cm)	84.5 ± 9.3
Arm span (cm)	158.6 ± 8.3
Risser sign (stage)	2.9 ± 1.1
Pelvic obliquity (°)	4.1 ± 3.0
Lumbar Cobb angle (°)	22.8 ± 12.5
Thoracic curve Cobb angle (°)	24.6 ± 11.6
Coronal balance (mm)	− 9.0 ± 13.5

Data are presented as frequency or mean ± standard deviation

Functional LLD (mm) was significantly greater than structural LLD (5.6 ± 5.0 vs. 0.2 ± 3.6 mm; *p* < 0.001). Additionally, no patients had a structural LLD ≥ 10 mm although 18 patients had a functional LLD ≥ 10 mm.

Functional and structural LLDs were significantly correlated with pelvic obliquity (*p* < 0.001 for both; Table 2). Functional LLD, but not structural LLD, was significantly correlated with the lumbar Cobb angle (*p* < 0.001 and *p* = 0.12, respectively). In addition, both functional and structural LLDs were not significantly correlated with thoracic Cobb angle (*p* = 0.43 and *p* = 0.68, respectively). Further, the mean coronal balance was − 9.0 ± 13.5 mm, and only one patient had a coronal balance > 40 mm.

**Table 2:** Pearson’s correlation coefficients between leg length deficiencies and spinopelvic alignment parameters

	Pelvic obliquity (*r*)	Lumbar Cobb angle (*r*)	Thoracic curve Cobb angle (*r*)
Functional LLD	0.69*	0.44*	0.09
Structural LLD	0.51*	0.17	− 0.05

*LLD* leg length discrepancy

*Statistically significant at *p* < 0.05

## Discussion

It is necessary to measure 3D leg length in the standing position in order to compare functional and structural LLDs under the same conditions. However, lower limb analyses rely on conventional radiography (2D view) or computed tomography in the decubitus position in clinical practice. Unfortunately, these modalities do not allow 3D analyses in the standing position [11]. Recently, an EOS 2D/3D system was developed that allows simultaneous PA and lateral 2D images of the whole body to be obtained in a calibrated environment, permitting the 3D reconstruction of spinal and lower limb bony structures via stereoradiography [7]. Guenoun et al. found that the EOS 3D modeling technique showed excellent inter- and intra-observer reliabilities, which were better than the reliabilities for 2D measurements [12]. In addition, several reports found that EOS is more accurate than both computed tomography scanography and conventional radiography [13, 14]. Therefore, the present study capitalized on this recent technological advancement to measure 3D structural LLDs, as well as functional LLDs, in patients with AIS. We revealed that a difference exists between functional LLDs and structural LLDs in patients with AIS. Furthermore, a functional LLD ≥ 10 mm was noted in 18 patients, while no patients had a structural LLD > 10 mm. Thus, although standing PA radiographs often show significant LLDs in patients with AIS at our clinical practice, the results of the present study revealed only a small amount of true LLDs in patients with AIS.

Furthermore, we assessed the correlations between LLDs and spinopelvic parameters in patients with AIS. Several previous studies have examined the relationship between the pelvis and the lower limbs/spine in terms of both anthropological and clinical problems [15]. There is evidence of pelvic abnormalities in patients with AIS. For example, Qiu et al. noted that standing PA radiographs show that the concave and convex ilia are not always symmetrical in patients with AIS in clinical practice [16]. The authors concluded that this phenomenon may be due to transverse pelvic rotation. In addition, Jones et al. reported that LLDs are more common in individuals with Marfan syndrome than in the general population and are associated with increased structural scoliosis [17]. The authors also stated that correlations between LLDs and scoliosis severity and convexity suggest that the two entities are related, although no causal relationship is clear.

Several studies have reported that structural LLDs cause pelvic obliquity in the frontal plane and lumbar scoliosis with convexity toward the shorter extremity [18, 19]. Scoliosis that develops due to LLDs is included within functional scoliosis. This type of scoliosis regresses completely or partially when its cause (i.e., LLD) is removed. Raczkowski et al. reported that minor LLDs cause pelvic obliquity in the frontal plane, which in turn causes scoliosis in the lumbar region [19]. These changes are believed to result from asymmetry of the spinal static and dynamic loads, as well as from intervertebral disc dislocation. However, in the present study, the structural LLD was very slight. Thus, it is considered that functional LLDs were caused by scoliosis. Recently, Pasha et al. reported a 3D analysis of spinopelvic alignment in patients with AIS. The authors concluded that the pelvic coronal tilt is significantly correlated with functional LLD. Relative spinopelvic alignment was suggested as a compensatory mechanism to maintain trunk equilibrium [20]. These conclusions are consistent with the present results regarding structural LLD and coronal balance.

In this study, functional and structural LLDs were significantly correlated with pelvic obliquity. However, the structural LLDs were so small that the correlations between structural LLD and pelvic obliquity can be ignored. Furthermore, functional LLD, but not structural LLD, was correlated with lumbar curve. Given that functional LLD was greater than structural LLD in AIS patients, structural LLDs probably do not influence scoliosis and pelvic obliquity. Rather, it seems likely that scoliosis affects functional LLD and pelvic obliquity. Since coronal balance was within the normal range in almost all patients, the difference between the functional and structural LLDs is suggestive of a compensatory mechanism for poor coronal balance in scoliosis through extension and flexion of the lower limbs.

Our study has a few limitations, including a small sample size. In addition, we did not add the length of the ankle joints or the hip joints to the leg length. However, we considered that the subjects are young and we think that the deformity changes of ankle joints and hip joints are almost negligible. Finally, to accurately measure leg length, the subjects stood by shifting the left and the right feet by several centimeters during EOS radiography. We thought that the difference is small and the measured value is not affected, but it is necessary to investigate in the future.

## Conclusions

This study revealed that patients with AIS have functional (apparent) LLD, but not significant structural (true) LLD. The correlation between lumbar Cobb angle and functional LLD indicates that the lumbar curve contributes to functional LLD; thus, the difference between functional and structural LLDs represents a compensatory mechanism involving extension and flexion of the lower limbs.

## Abbreviations

2D: Two-dimensional
3D: Three-dimensional
AIS: Adolescent idiopathic scoliosis
LLDs: Leg length discrepancies
PA: Posterior-anterior

## References

[1] Perdriolle R, Vidal J (1987). Morphology of scoliosis: three-dimensional evolution. Orthopedics.

[2] Humbert L, De Guise JA, Aubert B, Godbout B, Skalli W (2009). 3D reconstruction of the spine from biplanar X-rays using parametric models based on transversal and longitudinal inferences. Med Eng Phys.

[3] Raso VJ, Lou E, Hill DL, Mahood J, Moreau MJ, Durdle NG (1998). Trunk distortion in adolescent idiopathic scoliosis. J Pediatr Orthop.

[4] Gurney B (2002). Leg length discrepancy. Gait Posture.

[5] Sabharwal S, Zhao C, McKeon JJ, McClemens E, Edgar M, Behrens F (2006). Computed radiographic measurement of limb-length discrepancy. Full-length standing anteroposterior radiograph compared with scanogram. J Bone Joint Surg Am.

[6] Terry MA, Winell JJ, Green DW, Schneider R, Peterson M, Marx RG (2005). Measurement variance in limb length discrepancy: clinical and radiographic assessment of interobserver and intraobserver variability. J Pediatr Orthop.

[7] Dubousset J, Charpak G, Dorion I, Skalli W, Lavaste F, Deguise J (2005). A new 2D and 3D imaging approach to musculoskeletal physiology and pathology with low-dose radiation and the standing position: the EOS system. Bull Acad Natl Med.

[8] Gheno R, Nectoux E, Herbaux B, Baldisserotto M, Glock L, Cotten A (2012). Three-dimensional measurements of the lower extremity in children and adolescents using a low-dose biplanar X-ray device. Eur Radiol.

[9] O’Brien MF, Kuklo TR, Blanke KM, Lenke LG (2004). The spinal deformity study group radiographic measurement manual.

[10] Lenke LG, Betz RR, Harms J, Bridwell KH, Clements DH, Lowe TG (2001). Adolescent idiopathic scoliosis: a new classification to determine extent of spinal arthrodesis. J Bone Joint Surg Am.

[11] Chaibi Y, Cresson T, Aubert B, Hausselle J, Neyret P, Hauger O (2012). Fast 3D reconstruction of the lower limb using a parametric model and statistical inferences and clinical measurements calculation from biplanar X-rays. Comput Methods Biomech Biomed Engin.

[12] Guenoun B, Zadegan F, Aim F, Hannouche D, Nizard R (2012). Reliability of a new method for lower-extremity measurements based on stereoradiographic three-dimensional reconstruction. Orthop Traumatol Surg Res.

[13] Escott BG, Ravi B, Weathermon AC, Acharya J, Gordon CL, Babyn PS (2012). EOS low-dose radiography: a reliable and accurate upright assessment of lower-limb lengths. J Bone Joint Surg Am.

[14] Garner MR, Dow M, Bixby E, Mintz DN, Widmann RF, Dodwell ER (2016). Evaluating length: the use of low-dose biplanar radiography (EOS) and tantalum bead implantation. J Pediatr Orthop.

[15] Boulay C, Tardieu C, Bénaim C, Hecquet J, Marty C, Prat-Pradal D (2006). Three-dimensional study of pelvic asymmetry on anatomical specimens and its clinical perspectives. J Anat.

[16] Qiu XS, Zhang JJ, Yang SW, Lv F, Wang ZW, Chiew J (2012). Anatomical study of the pelvis in patients with adolescent idiopathic scoliosis. J Anat.

[17] Jones KB, Sponseller PD, Hobbs W, Pyeritz RE (2002). Leg-length discrepancy and scoliosis in Marfan syndrome. J Pediatr Orthop.

[18] Papaioannou T, Stokes I, Kenwright J (1982). Scoliosis associated with limb-length inequality. J Bone Joint Surg Am.

[19] Raczkowski JW, Daniszewska B, Zolynski K (2010). Functional scoliosis caused by leg length discrepancy. Arch Med Sci.

[20] Pasha S, Aubin CE, Sangole AP, Labelle H, Parent S, Mac-Thiong JM (2014). Three-dimensional spinopelvic relative alignment in adolescent idiopathic scoliosis. Spine (Phila Pa 1976).

